# Central Sensitization in Knee Osteoarthritis: Relating Presurgical Brainstem Neuroimaging and PainDETECT‐Based Patient Stratification to Arthroplasty Outcome

**DOI:** 10.1002/art.40749

**Published:** 2019-03-06

**Authors:** Anushka Soni, Vishvarani Wanigasekera, Melvin Mezue, Cyrus Cooper, Muhammad K. Javaid, Andrew J. Price, Irene Tracey

**Affiliations:** ^1^ University of Oxford Oxford UK; ^2^ University of Oxford, Oxford, UK, and University of Southampton Southampton UK

## Abstract

**Objective:**

The neural mechanisms of pain in knee osteoarthritis (OA) are not fully understood, and some patients have neuropathic‐like pain associated with central sensitization. To address this, we undertook the present study in order to identify central sensitization using neuroimaging and PainDETECT and to relate it to postarthroplasty outcome.

**Methods:**

Patients awaiting arthroplasty underwent quantitative sensory testing, psychological assessment, and functional magnetic resonance imaging (fMRI). Neuroimaging (fMRI) was conducted during punctate stimulation (n = 24) and cold stimulation (n = 20) to the affected knee. The postoperative outcome was measured using the Oxford Knee Score, patient‐reported moderate‐to‐severe long‐term pain postarthroplasty, and a range of pain‐related questionnaires.

**Results:**

Patients with neuropathic‐like pain presurgery (identified using PainDETECT; n = 14) reported significantly higher pain in response to punctate stimuli and cold stimuli near the affected joint (*P* < 0.05). Neural activity in these patients, compared to those without neuropathic‐like pain, was significantly lower in the rostral anterior cingulate cortex (*P* < 0.05) and higher in the rostral ventromedial medulla (RVM) during punctate stimulation (*P* < 0.05), with significant functional connectivity between these two areas (r = 0.49, *P* = 0.018). Preoperative neuropathic‐like pain and higher neural activity in the RVM were associated with moderate‐to‐severe long‐term pain after arthroplasty (*P* = 0.0356).

**Conclusion:**

The psychophysical and neuroimaging data suggest that a subset of OA patients have centrally mediated pain sensitization. This was likely due to supraspinally mediated reductions in inhibition and increases in facilitation of nociceptive signaling, and was associated with a worse outcome following arthroplasty. The neurobiologic confirmation of central sensitization in patients with features of neuropathic pain, identified using PainDETECT, provides further support for the investigation of such bedside measures for patient stratification, to better predict postsurgical outcomes.

## Introduction

Neuropathic pain is defined as pain caused by a lesion or disease of the somatosensory system 1. In contrast, nociceptive pain is defined as pain that arises from actual or threatened damage to non‐neural tissue and is due to the activation of nociceptors 1. Traditionally, pain in osteoarthritis (OA) was thought to be purely nociceptive, but screening tools such as the PainDETECT Questionnaire (PD‐Q) 2 have suggested a neuropathic component in some patients 3, 4, 5. Animal studies, symptom‐based assessments, quantitative sensory testing, and early neuroimaging studies show that central sensitization may be an important mechanism in a subgroup of patients, even in the absence of the structural lesion in the nervous system that is typically required in order to fulfill the definition of neuropathic pain 6, 7, 8. This type of pain might be more characteristic of nociplastic pain, a third category recently endorsed by the International Association of the Study of Pain, which acknowledges an abnormal pain state “characterized by clinical and psychophysical findings that suggest altered nociception, despite there being no clear evidence of actual or threatened tissue damage causing the activation of nociceptors or evidence for disease or lesion of the somatosensory system causing the chronic pain” 1, 9. However, non‐neural tissue is damaged in OA (meaning that the pain is nociceptive), so the nociplastic definition is not ideal either. Nonetheless, as clear lesions of the somatosensory system have not been identified in the context of OA, the term “neuropathic‐like pain” is used in the present report to describe patients in whom symptoms suggestive of neuropathic pain have been identified.

Central sensitization is defined as an amplification of neural signaling within the central nervous system that elicits pain hypersensitivity 10. It arises from a wide variety of underlying mechanisms ranging from sensitization within the spinal cord to signal amplification secondary to active descending pain facilitation pathways. The mechanisms by which central sensitization develops in OA and its impact on response to current treatment options remain unclear.

Neuroimaging provides a noninvasive objective method for measuring the central processing of pain in humans. Its utility in furthering our understanding of the pain mechanisms in patient populations and of suitable treatment options is increasingly accepted 11, 12. Previous neuroimaging studies in OA patients have demonstrated that both spontaneous and experimentally induced pain are associated with increased neural activity in brain areas involved in sensory discrimination 7, 13, 14, 15, 16 as well as with the affective and cognitive‐evaluative components of nociception 13, 14, 17. Furthermore, when compared to healthy controls, OA patients exhibited a disruption of the resting state default mode network 15 and a decrease in gray matter volume in areas such as the thalamus 16. Taken together, these findings suggest that both the structure and function of the brain are likely to be affected in patients with knee OA.

In patients with hip OA, neuroimaging results have also demonstrated the involvement of brainstem areas such as the periaqueductal gray (PAG), a component of the descending pain modulatory system (DPMS) 7. In that study, punctate stimulation in an area of referred pain in the OA patients was associated with increased activation in the PAG, when compared to healthy participants. Furthermore, patients with features of neuropathic pain, identified using the PD‐Q and psychophysical assessment, showed significantly greater activation within the PAG compared to those with a low PD‐Q score. This provided direct evidence of central sensitization in patients with OA, linking activity in the DPMS to neuropathic‐like features.

Cortical and subcortical brain areas are known to modulate nociception by interacting with the midbrain and medullary structures that form the DPMS 18. This is a well‐characterized network that regulates nociceptive processing in the dorsal horn via inhibitory and facilitatory influences 12, 19. The midbrain PAG mainly exerts its effect through the rostral ventromedial medulla (RVM), which is thought to represent the final relay in descending modulation from supraspinal sites 20. The RVM can both inhibit and facilitate pain 21, 22, and it is thought that an imbalance between the inhibitory and facilitatory tone of the DPMS may contribute to an abnormal chronic pain state 12. Cortical and subcortical areas of the brain, including the anterior cingulate cortex, amygdala, insula, and hypothalamus, are also involved in pain modulation via the DPMS. This link is likely to explain how other centrally mediated factors such as sleep, cognition, mood, and placebo effects influence the experience of pain 23.

Neuroimaging studies in humans with central sensitization have shown the involvement of the PAG, the adjacent nucleus cuneiformis (NCF), and the mesopontine reticular formation, which are also major sources of input to the RVM 24, 25. Furthermore, preclinical work has shown that the central sensitization seen in conjunction with neuropathic features of pain in OA is partly mediated by descending modulation, and that pain relief was achieved by blocking descending pain facilitatory pathways from the RVM 21, 22, 26. These findings are consistent with a substantial body of work that has demonstrated that central nervous system nociceptive processing is altered in patients with other musculoskeletal conditions such as fibromyalgia 27, 28. Interestingly, the coexistence of characteristics of fibromyalgia (suggestive of augmented central nervous system pain processing) in patients with OA has been associated with a poorer outcome following arthroplasty 29.

In this study, we investigated the neural correlates of the features of neuropathic pain (as measured using the modified PD‐Q [mPD‐Q]), compared to nociceptive pain, in knee OA. Furthermore, we examined the relationship between central sensitization (identified using functional magnetic resonance imaging [fMRI]) and the outcome following knee replacement surgery, a treatment option that only addresses the peripheral nociceptive drive for pain. Based on previous literature and our own work, we chose to specifically examine the PAG, NCF, and RVM. We hypothesized that patients with features of neuropathic pain would show higher activity in response to a painful stimulus in these brainstem regions and have a worse outcome following surgery, compared to those with nociceptive pain.

## Patients and Methods

#### Patients

Participants recruited to the Evaluation of Perioperative Pain in Osteoarthritis of the Knee (EPIONE) Study, a prospective cohort study of patients with primary OA who were awaiting primary knee replacement surgery 30, were invited to take part in this neuroimaging substudy. Patients were recruited from the Nuffield Orthopaedic Centre in Oxford, UK. The local ethics committee approved the study (National Research Ethics Service‐South Central‐Oxford B, 09/H0605/76), and written consent was obtained from each participant. Sample size in our study was based on a previous fMRI study using a similar stimulus paradigm 7. In that study, there was a significant difference in the mean ± SD percentage of neural activity signal evoked by punctate stimuli between patients with neuropathic pain–like features (0.35 ± 0.25) and those with nociceptive pain (0.98 ± 0.40). Therefore, to detect a statistically significant result with a *P* value of less than 0.05 and a probability of 80%, the estimated sample size per group is 9.

#### Psychophysical assessment

Validated questionnaires were used to assess psychological characteristics and sleep disturbances. These included the State‐Trait Anxiety Inventory 31, the Pain Catastrophizing Scale 32, the Tampa Scale for Kinesiophobia 33, the Hospital Anxiety and Depression Scale 34, and the Pittsburgh Sleep Quality Index 35.

#### Oxford Knee Score

The primary outcome measure following surgery was the Oxford Knee Score (OKS) 36, which measures 3 symptom domains: pain, stiffness, and functional disability, in relation to the knee. It has been shown that an OKS of ≥37 can be used to identify patients who are more likely to have achieved an acceptable state of postoperative functioning 37. Pain and function subscales, which can be calculated using original data from the OKS, have also been defined and validated 38. These subscales are scored from 0 (best possible score, least severe symptoms) to 100 (worst possible score, most severe symptoms). OKS results at 12 months after surgery were collected as part of a postal questionnaire; participants who did not initially respond were sent 2 postal reminders. The proportion of patients (in each pain group) with moderate‐to‐severe long‐term pain after arthroplasty was used as a secondary outcome measure. Moderate‐to‐severe long‐term postoperative pain was measured 12 months after surgery using the visual analog scale in the short form of the McGill Pain Questionnaire 39, 40 and defined by an average pain severity score of ≥3 for the preceding week 41.

#### Quantitative sensory testing (QST)

QST measures including cold detection and cold pain thresholds, as well as mechanical pain threshold in alignment with the standard research protocol for QST 42, were conducted prior to the scan session. Mechanical punctate pain intensity was also measured using a 512‐mN punctate probe. For this assessment, a single punctate pain stimulus was delivered over the medial joint line of the affected joint, and the participant was asked to rate the intensity of the pain stimulus on a numeric rating scale (0–10), with 0 indicating that the stimulus was not at all sharp and 10 indicating the sharpest imaginable pain. This was repeated 3 times, and the average pain rating was calculated by taking the arithmetic mean of the 3 readings. Patients were also asked to rate the severity of their current knee pain using a visual analog scale just before commencing the scanning experiment.

#### Functional MRI scanning protocol

Brain images were acquired using a Siemens Magnetom Verio 3.0T MRI system and a 32‐channel head coil. Scan data were acquired during cold stimulation, punctate stimulation, and rest. Participants completed perception ratings at the end of each paradigm (see Supplementary Methods, on the *Arthritis & Rheumatology* web site at http://onlinelibrary.wiley.com/doi/10.1002/art.40749/abstract).

#### Blood oxygen level–dependent (BOLD) imaging analysis

Prior to analysis, the BOLD fMRI data for those with left‐sided knee pain were flipped so that the left–right orientation was comparable across the group. Functional MRI data processing was carried out using an fMRI Expert Analysis Tool, version 6.0 (www.fmrib.ox.ac.uk/fsl). Details on how statistical images were generated to identify significant brain activity evoked by cold pain and punctate paradigms are available in Supplementary Methods and Supplementary Figure 1 (http://onlinelibrary.wiley.com/doi/10.1002/art.40749/abstract). In addition to whole brain analyses, regions of interest analyses were also conducted for the areas in the brainstem, defined a priori (Supplementary Methods).

Post hoc analyses were conducted to further investigate the association of clinical measures of neuropathic pain severity with changes in the BOLD signal in brain areas found to show significantly different levels of activation between the nociceptive and neuropathic‐like pain groups. The parameter estimates for these regions were compared to the postoperative outcome OKS and the presence of moderate‐to‐severe long‐term pain at 12 months after arthroplasty (Supplementary Methods).

#### Seed‐based functional connectivity

In order to further extend the findings of the stimulus‐evoked fMRI data, resting state data were used to interrogate connectivity between the RVM and the rostral anterior cingulate cortex (rACC). Considering the emerging evidence suggesting that preexisting aberrant connectivity in the reward system, especially involving the nucleus accumbens (NA), may contribute to chronic pain and an inability to derive relief from pain‐relieving interventions 43, 44, 45, an additional post hoc analysis of connectivity between the RVM and NA was conducted (Supplementary Methods, http://onlinelibrary.wiley.com/doi/10.1002/art.40749/abstract).

#### Data analysis

The mPD‐Q was used to subclassify patients according to established cutoff values for nociceptive pain, unclear pain, and neuropathic pain 2. For the purposes of comparing those with purely nociceptive clinical pain to those with features of neuropathic pain, the unclear pain group was combined with the neuropathic pain group, which is referred to as the neuropathic‐like pain group. This approach is consistent with those from previous studies 3, 8 and ensures that patients with possible neuropathic pain are included. The differences in psychophysical characteristics between the nociceptive and neuropathic‐like pain groups at baseline were investigated using Student's *t*‐test for normally distributed data, Wilcoxon‐Mann‐Whitney test for non‐normally distributed data, and Fisher's exact test for categorical data. The Wilcoxon‐Mann‐Whitney test was used to investigate differences in OKS between the 2 groups postoperatively. Fisher's exact test was used to determine differences in the proportion of patients who reported moderate‐to‐severe long‐term pain after arthroplasty and those who achieved patient‐acceptable symptom state at 12 months postsurgery.

## Results

Twenty‐six participants were enrolled in the study. One participant was excluded from all analyses due to excess motion artifact, and a second participant was excluded due to incidental structural abnormality precluding adequate registration. Of the remaining 24 participants, the cold paradigm was not completed in 4 participants due to technical problems with the thermode.

#### Psychophysical characteristics

Ten patients met the criteria for nociceptive pain, using standard cutoff criteria for the mPD‐Q. The remaining 14 patients were included in the neuropathic‐like pain group. Although in the neuropathic‐like pain group there were trends toward younger age, higher proportion of female subjects, shorter duration of knee pain, and more severe symptoms prior to surgery, none of these differences reached statistical significance (Table 1). The data on psychological measures demonstrated significant increases in fear of movement and pain catastrophizing in the neuropathic‐like pain group compared to the nociceptive group.

**Table 1:** Preoperative characteristics of participants in the neuroimaging substudy, divided according to the presence or absence of neuropathic pain features^a^

	Nociceptive pain group (n = 10)	Neuropathic‐like pain group (n = 14)
Clinical features		
Age, mean + SD years	70 ± 7	67 ± 10
Female, no. (%)	3 (30)	8 (57)
Right knee affected, no. (%)	3 (30)	7 (50)
Duration of pain, median (IQR) months	60 (24, 108)	24 (18, 60)
OKS, mean + SD (range 0–48)	20.5 ± 6.7	17.0 ± 6.5
OKS pain subscale, mean + SD (range 0–100)	69.2 ± 12.0	74.8 ± 11.2
OKS function subscale, mean + SD (range 0–100)	61.2 ± 13.1	67.1 ± 11.2
Psychological features		
HAD anxiety, mean + SD (range 0–21)	6.4 ± 4.5	8.0 ± 3.4
HAD depression, mean + SD (range 0–21)	6.9 ± 2.3	7.3 ± 3.0
STAI state anxiety, mean + SD (range 20–80)	31.8 ± 16.2	39.9 ± 13.5
STAI trait anxiety, mean + SD (range 20–80)	31.6 ± 12.9	38.1 ± 13.7
PCS, median (IQR) (range 0–52)	11 (3, 14)	18 (7, 30)^b^
TSK, mean + SD (range 17–68)	33.4 ± 5.3	39.2 ± 4.4^b^
PSQI, mean + SD (range 0–21)^c^	8.6 ± 1.7	10.7 ± 4.4
QST measures at the knee		
Mechanical pain threshold, median (IQR) mN	96.0 (32.0, 128.0)	32.0 (25.4, 101.6)
Sharpness rating to 512‐mN probe, mean + SD (range 0–10)	4.5 ± 2.4	5.1 ± 2.8
Cold detection threshold, median (IQR) °C	27.7 (27.1, 28.3)	28.7 (27.8, 29.6)
Cold pain threshold, median (IQR) °C	10 (10, 12.0)	20.4 (10, 23.1)^b^
Stimulus ratings in the scanner		
Unpleasantness of cold stimuli, median (IQR) (range 0–100)	0.0 (0.0, 0.0)	4.5 (0.0, 9.5)
Pain with cold stimuli, median (IQR) (range 0–100)	0.0 (0.0, 24.0)	3.5 (0.0, 24.0)
Sharpness of punctate stimuli, median (IQR) (range 0–100)	0.0 (0.0, 27.0)	17.5 (10.0, 36.0)
Unpleasantness of punctate stimuli, median (IQR) (range 0–100)	0.0 (0.0, 10.0)	11.0 (5.0, 20.0)^b^
Knee pain ratings in the scanner		
Pain severity immediately prior to experiment, median (IQR) (range 0–100)	0.0 (0.0, 20.0)	20.0 (0.0, 50.0)
Pain severity after cold stimuli, median (IQR) (range 0–100)	3.5 (0.0, 10.5)	3.0 (0.0, 18.5)
Pain severity after punctate stimuli, median (IQR) (range 0–100)	0.0 (0.0, 0.0)	5.5 (0.0, 27.0)^b^

a The preoperative modified PainDETECT questionnaire score was used to subdivide patients by nociceptive pain (<13) and neuropathic pain (>13), and the statistical significance of differences between groups was assessed. IQR = interquartile range; OKS = Oxford Knee Score; HAD = Hospital Anxiety and Depression Scale; STAI = State‐Trait Anxiety Inventory; PCS = Pain Catastrophizing Score; TSK = Tampa Scale of Kinesiophobia; QST = quantitative sensory testing.

b *P* < 0.05 versus nociceptive pain group.

c Measures of the Pittsburgh Sleep Quality Index (PSQI) were only available for 8 and 12 participants in the nociceptive and neuropathic‐like pain groups, respectively.

For readings obtained with the patient outside the scanner, sensitivity to cold pain was significantly higher in the neuropathic‐like pain group compared to the nociceptive pain group (*P* < 0.05). The remaining QST parameters showed a nonsignificant trend toward increased sensitivity in the neuropathic‐like pain group compared to the nociceptive pain group.

For readings obtained with the patient inside the scanner, the neuropathic‐like pain group reported significantly greater levels of unpleasantness in response to the punctate stimuli (*P* < 0.05). The neuropathic‐like pain group also tended to report higher scores for the other ratings, but these did not reach statistical significance. Finally, the neuropathic‐like pain group reported significantly greater knee pain immediately after the punctate paradigm, compared to the nociceptive pain group (*P* < 0.05).

#### Functional MRI results

In the punctate paradigm (n = 24), the stimuli evoked increased brain activity bilaterally in the secondary somatosensory cortex, anterior and posterior insula, and supplementary motor area, as well as in the mid–anterior cingulate cortex (Supplementary Figure 2, http://onlinelibrary.wiley.com/doi/10.1002/art.40749/abstract). Deactivation was seen in the precuneous and contralateral primary motor cortex. The cold paradigm (n = 20) was associated with activation in the following areas bilaterally: secondary somatosensory cortex, caudate, thalamus, cerebellum, and contralateral insula and putamen (Supplementary Figure 2). Deactivation during the cold paradigm was observed in the precuneous and anterior paracingulate gyrus.

**Figure 1:**
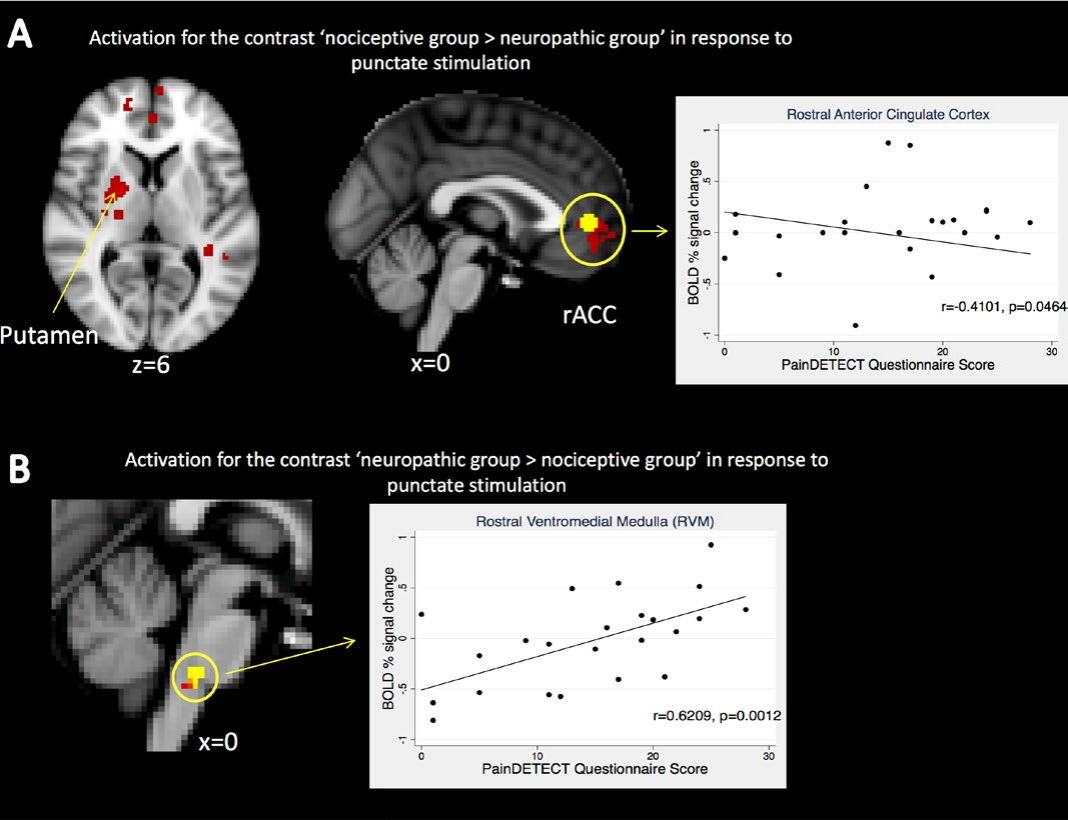
Whole‐brain analysis and region of interest analysis results of punctate stimulation. **A**, Mixed‐effects, whole‐brain analysis comparing responses to punctate stimulation between the neuropathic‐like pain group (n = 14) and the nociceptive pain group (n = 10). Correlation between the change in blood oxygen level–dependent (BOLD) signal activity in the rostral anterior cingulate cortex (rACC) and the severity of neuropathic‐like pain symptoms is shown. Significantly increased activity in the nociceptive pain group compared to the neuropathic‐like pain group is indicated (red), and a functional mask was generated using a 5‐mm sphere from the peak voxel of activation in the rACC cluster (yellow). There were no areas in which activation was significantly higher in the neuropathic‐like pain group than in the nociceptive pain group. Whole‐brain analyses were corrected for multiple comparisons (Z score >2.3, *P* < 0.05). B, Region of interest analysis comparing responses (n = 24) to punctate stimulation between the neuropathic‐like pain group (n = 14) and the nociceptive pain group (n = 10). Correlation between the change in BOLD signal activity in the rostral ventromedial medulla (RVM) and the severity of neuropathic‐like pain symptoms is shown. Region of interest test statistics were generated from a generalized linear model design, thresholded using threshold‐free cluster enhancement. *P* < 0.05. Images are displayed in radiologic convention with Montreal Neurological Institute coordinates given.

For the punctate paradigm, the nociceptive pain group (n = 10) demonstrated significantly higher activation in the rACC and the ipsilateral putamen using whole‐brain comparisons, compared to the neuropathic‐like pain group (n = 14) (Figure 1A). The change in BOLD activation in the rACC showed a significant inverse relationship with the severity of neuropathic‐like pain features, measured using the mPD‐Q (r = −0.4101, *P* < 0.05) (Figure 1A). There were no areas in which activation was significantly higher in the neuropathic‐like pain group than in the nociceptive pain group. The small sample size (7 patients in the nociceptive pain group and 13 in neuropathic‐like pain group) precluded meaningful subgroup analysis for the cold paradigm.

Region of interest analyses revealed increased activation in the ipsilateral NCF (*P* < 0.05) (Supplementary Figure 3, http://onlinelibrary.wiley.com/doi/10.1002/art.40749/abstract) and RVM (*P* < 0.05) (Figure 1B) in the neuropathic‐like pain group, compared to the nociceptive pain group, during punctate stimulation. There was no significant difference in activation in the PAG. There was no significant association between BOLD signal change and the mPD‐Q score in the NCF (Supplementary Figure 3). The change in BOLD activation in the RVM was significantly and strongly positively correlated with the mPD‐Q score (r = 0.6209, *P* = 0.0012) (Figure 1B).

#### Connectivity results

**Figure 2:**
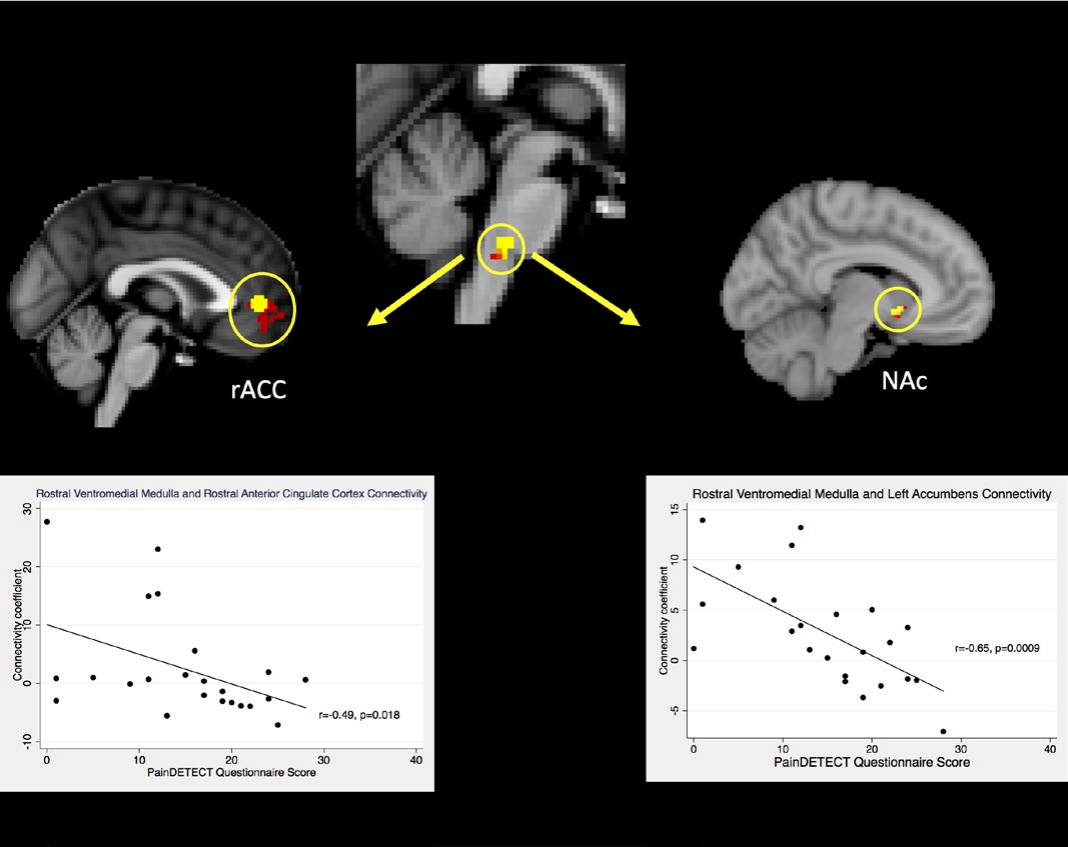
Rostral ventromedial medulla seed‐based functional connectivity analysis. Correlation between the connectivity coefficient, for the rACC and NAc, with severity of neuropathic‐like pain symptoms (n = 23) is shown. Test statistics were generated from a generalized linear model design and thresholded using threshold‐free cluster enhancement. *P* < 0.05. rACC = rostral anterior cingulate cortex; NAc = nucleus accumbens.

Whole‐brain analysis did not reveal any significant differences in connectivity with the RVM or rACC between the nociceptive pain group and neuropathic‐like pain group. Region of interest analysis demonstrated that connectivity between the RVM and the rACC was greater in the nociceptive pain group than in the neuropathic‐like pain group with the RVM seed‐based analysis only (Figure 2). In addition, region of interest analysis showed increased connectivity between the RVM and contralateral NA in the nociceptive pain group, compared to the neuropathic‐like pain group (Figure 2).

#### Clinical and psychological features 12 months postsurgery

**Figure 3:**
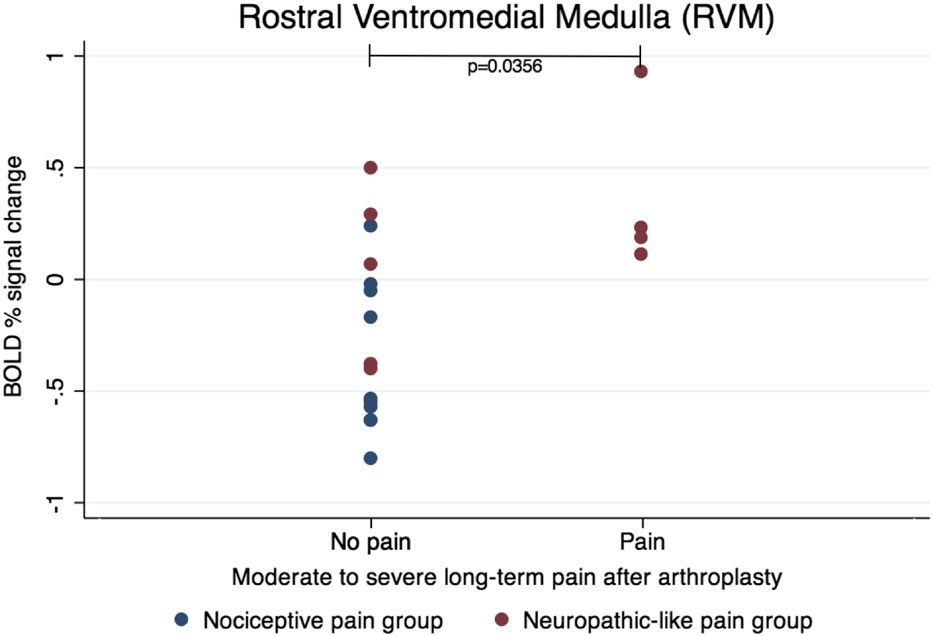
Relationship between functional magnetic resonance imaging activation in the rostral ventromedial medulla prior to surgery and clinical outcome at 12 months (n = 19). BOLD = blood oxygen level–dependent.

Long‐term follow‐up data were available for 19 patients. All of the clinical and psychological features showed significant improvement compared to baseline (*P* < 0.05), except for state and trait anxiety, pain catastrophizing, and sleep disturbance. Anxiety and pain catastrophizing were found to be significantly worse in the neuropathic‐like pain group, compared to the nociceptive pain group. In the neuropathic‐like pain group, there was a nonsignificant trend toward worse clinical symptom severity, which was assessed using the OKS and the proportion of patients achieving a patient‐acceptable symptom state. However, the neuropathic‐like pain group, as defined presurgically, did have a significantly higher proportion of patients with moderate‐to‐severe long‐term pain after arthroplasty, compared to the nociceptive pain group (Table 2). Furthermore, patients with moderate‐to‐severe long‐term pain after arthroplasty had significantly higher BOLD signal change in the RVM prior to surgery (median −0.38 [interquartile range −0.58, 0.07]), compared to those who did not report long‐term pain after arthroplasty (median 0.21 [interquartile range 0.14, 0.58]) (*P* = 0.0356) (Figure 3). There was no significant relationship between BOLD signal change in the rACC and long‐term pain after arthroplasty (Supplementary Figure 4, http://onlinelibrary.wiley.com/doi/10.1002/art.40749/abstract), and there was no significant association between BOLD signal change in the RVM or rACC during punctate stimulation and OKS results 12 months postsurgery.

**Table 2:** Twelve‐month postoperative characteristics of participants in the neuroimaging substudy, divided according to the presence or absence of neuropathic pain features^a^

	Nociceptive pain group (n = 10)	Neuropathic‐like pain group (n = 9)
Clinical features		
OKS, median (IQR) (range 0–48)	46.0 (40.0, 47.0)	40.0 (33.0, 48.0)
OKS pain subscale, median (IQR) (range 0–100)	26.0 (24.0, 32.0)	36.0 (20.0, 52.0)
OKS function subscale, median (IQR) (range 0–100)	20.0 (20.0, 28.6)	31.7 (21.5, 37.2)
Patient‐acceptable symptom state, no. (%)	9 (90)	5 (56)
Moderate‐to‐severe long‐term pain after arthroplasty, no. (%)	0 (0)	4 (44)^b^
Psychological features		
HAD anxiety, median (IQR) (range 0–21)	0.5 (0.0, 2.0)	3.0 (1.0, 7.0)^b^
HAD depression, median (IQR) (range 0–21)	1.0 (0.0, 3.0)	1.0 (0.0, 7.0)
STAI state anxiety, mean + SD (range 20–80)	24.0 ± 10.2	33.0 ± 15.8
STAI trait anxiety, mean + SD (range 20–80)	28.0 ± 5.5	33.9 ± 12.7
PCS, median (IQR) (range 0–52)	5 (0, 6)	14 (2, 17)^b^
PSQI, mean + SD (range 0–21)	7.8 ± 2.9	8.7 ± 4.3

a The preoperative PainDETECT questionnaire score was used to subdivide patients by nociceptive pain (<13) and neuropathic pain (>13), and the statistical significance of differences between groups was assessed. OKS = Oxford Knee Score; IQR = interquartile range; HAD = Hospital Anxiety and Depression Scale; STAI = State‐Trait Anxiety Inventory; PCS = Pain Catastrophizing Score; PSQI = Pittsburgh Sleep Quality Index.

b *P* < 0.05 versus nociceptive pain group.

## Discussion

The main finding of this preliminary study is that patients awaiting arthroplasty for knee OA who have features of neuropathic pain (identified using the mPD‐Q) demonstrated psychophysical and functional imaging evidence of centrally mediated pain sensitization, compared to OA patients with nociceptive pain. The neuropathic‐like pain group exhibited significantly lower levels of activation in the rACC (Z score >2.3, *P* < 0.05) and higher levels of activation in the RVM (*P* = 0.00182) and ipsilateral NCF (*P* = 0.02962) in response to punctate stimulation of the affected knee, compared to those with features of nociceptive pain. In addition, the resting state data showed increased connectivity between the RVM and rACC, as well as between the RVM and NA, in the nociceptive pain group compared to the neuropathic‐like pain group. Psychophysically, the neuropathic‐like pain group had significantly higher sensitivity to cold and punctate stimuli, as well as significantly higher levels of pain catastrophizing and kinesiophobia, prior to surgery.

Following knee replacement surgery, there was a trend toward worse outcomes in the neuropathic‐like pain group, with a significantly higher proportion of patients experiencing moderate‐to‐severe long‐term pain after arthroplasty. Moreover, punctate stimuli–evoked RVM activation prior to surgery was significantly higher in patients reporting moderate‐to‐severe long‐term pain after arthroplasty compared to those who did not. These findings may be related to the well‐established fact that pain severity prior to surgery is an important predictor of persistent pain after total knee arthroplasty 46. In the current study, patients with neuropathic‐like pain reported higher levels of pain and disability prior to surgery but the difference was not statistically significant, possibly due to small sample sizes. The current study, in attempting to stratify patients by different mechanisms for pain, provides additional insight into why some patients, who were also likely to have higher preoperative pain severity, had a higher probability of experiencing unsatisfactory pain relief from arthroplasty.

The rACC is an important cortical area involved in the descending inhibitory control of pain, which works by recruiting an antinociceptive subcortical network, including the amygdalae and PAG 47. Its role in regulating pain has been most extensively investigated in the context of placebo analgesia, where the effect is mediated by the endogenous opioid system via μ‐opioid receptor activation in specific brain regions, including the rACC 48. The current study shows that patients with knee OA who demonstrated increased rACC activation in response to punctate stimulation were less likely to report features of neuropathic‐like pain with respect to their clinical knee pain. The mechanism underlying the differences in the manifestation of the same clinical condition may therefore be associated with the differential ability to successfully engage the endogenous inhibitory system in patients from the nociceptive pain group, compared to the neuropathic‐like pain group. Although the role of the rACC in patients with knee OA had not been previously reported, the current results are consistent with previous observations in fibromyalgia, where reduced structural and functional connectivity in the rACC was demonstrated when compared to control participants 49 and interpreted as a dysfunction in descending inhibition.

The NCF is known to be part of the descending pain modulatory system, and a previous study of experimentally induced central sensitization in healthy participants showed increased activation in the contralateral NCF during hyperalgesia 24. Findings from the current study support a similar involvement of the NCF in the context of clinical pain sensitization secondary to knee OA.

The RVM is known to receive input from the PAG and adjacent NCF and is considered to be the final relay point for the descending supraspinal signals, before modifying incoming nociceptive signals in the dorsal horn of the spinal cord 11. The descending modulation of spinal cord function was originally thought to involve only inhibitory mechanisms, but over time the role of facilitatory effects on nociceptive processing has been recognized, demonstrated in the imaging of humans in injury models, and even shown to be modifiable by analgesics 19, 21, 22, 24, 50, 51, 52. As such, the RVM is highly likely to be involved in chronic pain development and maintenance 51.

In the current study, increased activation in the RVM in patients with neuropathic‐like pain, compared to those with nociceptive pain, may reflect increased activity in RVM on‐cells, resulting in descending facilitation similar to what was previously found in hip OA 7 and in our earlier studies that focused on imaging of centrally sensitized states 24, 25. Functional MRI data alone do not allow us to distinguish between facilitatory and inhibitory activity in the RVM, and it is possible that the increased activity reflects increased inhibitory drive in response to the greater pain severity associated with the presence of neuropathic‐like pain. However, the former hypothesis is somewhat supported by the fact that the mPD‐Q score was significantly and positively correlated with the level of RVM activity, as well as by the psychophysical differences that demonstrated heightened fear of movement, pain catastrophizing, punctate unpleasantness score, and joint pain after punctate stimulation in the neuropathic‐like pain group compared to the nociceptive group (Table 2). Furthermore, our recent work in diabetic painful peripheral neuropathy emphasizes the facilitatory role of DPMS brainstem nuclei 53.

In accordance with the dual capability of the RVM to inhibit and facilitate pain processing, the higher rate of connectivity between the RVM and rACC in the nociceptive pain group suggests that the RVM in these patients is likely to exert an inhibitory effect on pain. Additionally, before surgery, these patients had higher connectivity between the RVM and NAC (a key structure of the reward processing system), and it was evident that they had better pain relief results postoperatively. This is consistent with the emerging concept that an intact reward system may be important for experiencing pain relief 45. However, this post hoc finding requires further validation in a separate study.

The current study did not demonstrate any significant differences in PAG activation between the 2 patient groups. This is surprising, given the previous findings in patients with hip OA 7 and knowledge about the functional connectivity of the rACC and the descending modulation of pain. It is possible that the involvement of the PAG in more than 1 function and its multiple connections, in conjunction with its relatively small size 54, contributed to a lack of significant difference between the patient groups in this study. Further studies using ultra‐high‐field imaging or enhanced acquisition sequences to enable functional neuroanatomical dissection of the PAG into its constituent components, as we did for the diabetic painful neuropathy study 53, should also help identify differences in pain‐related PAG function that may exist between patient groups.

The main strength of the current study is that it recruited patients with clinically homogeneous disease severity, in that they were all deemed to be appropriate candidates for knee replacement surgery. Moreover, the neuroimaging data were related to behavioral and QST measures. The main limitation of the study was the use of the mPD‐Q to stratify patients, as the questionnaire was designed to measure more broadly neuropathic pain rather than specifically and only central sensitization. Since the design of the study, other tools have been developed to specifically identify features of centralized pain, such as the modified 2010 American College of Rheumatology Preliminary Diagnostic Criteria for Fibromyalgia 55 and the Central Sensitization Inventory 56.

It may also be beneficial to compare the responses in the nociceptive pain group to the neuropathic‐like pain group without inclusion of the unclear pain group in the latter. Unfortunately, recruitment to a study like this is challenging due to the demographic and the potential for contraindications with MR. The sample size (while adequately powered) was not sufficient to conduct an analysis of arthroplasty outcome data, as only 19 patients returned postsurgery; this is something we would like to address in future studies. The relatively small sample size might also have contributed to the lack of a significant difference in postoperative OKS results between groups, especially as data from 2 larger patient cohorts confirm the difference in outcome between patients stratified by nociceptive pain and neuropathic‐like pain as both clinically and statistically significant 30. Nonetheless, the overall proportion of patients with unfavorable long‐term pain postarthroplasty, reported here, is reassuringly similar to the data reported in the literature 57.

Finally, what is not clear from this study or previous studies is whether changes in imaging findings are related to the pain or are the consequence of pain‐related biomechanical alterations due to musculoskeletal damage causing changes in gait, lifestyle, activity levels, etc. Attempts to decipher this by imaging patients after successful arthroplasty do show normalization of imaging findings 16, 58; however, it may be that pain relief facilitated improvement in biomechanics that largely explained the brain imaging changes. A similar line of reasoning supports recent work redefining phantom limb pain 59.

In summary, this preliminary study furthers our understanding of the underlying neurobiologic mechanisms in patients with knee OA who have clinical features suggestive of neuropathic pain. Explorative work has suggested that preoperative PD‐Q scores independently predict postoperative pain intensity 60. Current neuroimaging data suggest that this may be due to both reduced descending inhibitory mechanisms and increased supraspinal facilitation of nociceptive signals in the dorsal horn. The neurobiologic suggestion of central sensitization in patients with features of neuropathic pain, identified using the mPD‐Q, provides further support for investigation of stratified patient groups in order to better predict the outcome following surgery. Further work is needed to confirm the findings of this small‐scale study and to determine the optimal method for identifying the patient group most likely to have more complex underlying pain mechanisms, such as central sensitization. Clarification of the terminology used in this scenario (i.e., in which there is abnormal nociceptive processing in the absence of a structural lesion of the somatosensory system) will also be critical as this body of research progresses, in addition to enabling successful translation to the clinical setting. In the future, it may be possible to use this information to potentially guide the use of drug therapy and behavioral treatments to specifically target this mechanism in order to improve overall arthroplasty‐related treatment outcomes.

## Author Contributions

All authors were involved in drafting the article or revising it critically for important intellectual content, and all authors approved the final version to be published. Dr. Soni had full access to all of the data in the study and takes responsibility for the integrity of the data and the accuracy of the data analysis.

### Study conception and design

Soni, Cooper, Javaid, Price, Tracey.

### Acquisition of data

Soni, Wanigasekera, Mezue.

### Analysis and interpretation of data

Soni, Wanigasekera, Mezue, Javaid, Price, Tracey.

## Supporting information

Supplementary Figure 1
