# Supplementary material for: Central Sensitization in Knee Osteoarthritis: Relating Presurgical Brainstem Neuroimaging and PainDETECT‐Based Patient Stratification to Arthroplasty Outcome
Source: Arthritis Rheumatol. 2019 Mar 6;71(4):550–60. doi: 10.1002/art.40749 (PMC6430421; doi:10.1002/art.40749)
Supplement: Supplementary file 1 — Supplementary Figure 1 [file ART-71-550-s001.docx]

**Appendix**

**fMRI scanning protocol**

Brain images were acquired using a 3.0T Siemens MAGNETOM® Verio Magnetic Resonance Imaging System and a 32-channel head coil. A whole-brain gradient echo-planar imaging sequence was used to acquire blood oxygenation level–dependent (BOLD) functional images. For functional scans the parameters were: echo time (TE) of 30 msec, 46 contiguous 3-mm–thick slices, field of view 192x192mm, voxel size 3.0 x 3.0 x 3.0 mm^3^ and repetition time (TR) of 3000msec. After the BOLD imaging T-1 weighted structural scans, 1 x 1 x 1mm^3^, were acquired for registration and overlay of brain activation.

Physiological noise data was collected using respiratory bellows and a pulse oximeter, together with volume triggers from the scanner. All data was recorded using an MP150 system (BIOPAC Systems, Inc., Goleta, CA) at a sample rate of 50Hz. B_0_ field maps were also acquired to correct for regions of field inhomogeneity.

**Stimulation paradigm**

The fMRI scan paradigm is summarised in Figure 1. Cold stimuli were generated using 30 x 30 mm ATS thermode (Medoc, Ramat Yishai, Israel). Eight stimuli at 15^o^C, each of 14-second duration were applied to the medial joint line of the affected knee in a block design with a rest period between stimuli. This inter-stimulus interval (ISI) was jittered with a minimum of 90 seconds. Mechanical punctate stimuli were generated using a custom-made weighted pinprick simulator, which exerted a force of 512mN (MRC Systems). 15 stimuli were applied to the anterior aspect of the lower limb of the affected knee, at least 5cm distal to the tibial tuberosity. Each stimulus, or event, was separated by a jittered ISI of at least 15 seconds. This event-related design allows the haemodynamic response for each individual stimulus to be estimated. Participants were instructed to keep their eyes open and fixate on a cross during stimulation.

Participants completed perception ratings at the end of the cold and punctate stimulation paradigms. This was done using a computerized VAS displayed on a projector screen, which was visible whilst in the scanner. For both paradigms participants were asked to rate how unpleasant stimuli had felt on average. The anchor statements were ‘not unpleasant’ and ‘extremely unpleasant’. After the cold paradigm, participants were asked to rate how painful the stimuli had felt on average. The anchor statements for this rating were ‘not painful’ and ‘extremely painful’. The corresponding rating after the punctate paradigm captured the average sharpness of the painful stimuli, with the anchor statements ‘not sharp’ and ‘extremely sharp’. The final rating for each paradigm recorded the intensity of any ongoing knee pain attributed to the patients’ usual arthritis pain, using the anchor statements ‘not painful’ and ‘extremely painful’. The ratings were collected using Presentation software (version 16.0; Neurobehavioral Systems, Albany, CA) and a button box controlled by the participant in the scanner. During the same scanner session, imaging data were also collected for 10 minutes while resting immediately before and after exercise induced pain states.

Figure 1 Illustration of the scanning experimental paradigm performed by the study participants

*Questionnaires used to assess the psychological characteristics of the participants included: Hospital Anxiety and Depression Scale; STAI=State-Trait Anxiety Inventory; Pain Catastrophising Score; Pittsburgh Sleep Quality Index and Tampa scale of kinesophobia. CDT=cold detection threshold, CPT=cold pain threshold, MPT=mechanical pain threshold; OKS=Oxford Knee Score.

**BOLD imaging analysis**

Registration to high-resolution structural and standard space images was carried out using FLIRT. Registration from high resolution structural to standard space was then further refined using FNIRT nonlinear registration (1).

The following pre-statistics processing was applied; motion correction using MCFLIRT; non-brain removal using BET; spatial smoothing using a Gaussian kernel of FWHM 5mm; grand-mean intensity normalisation of the entire 4D dataset by a single multiplicative factor; highpass temporal filtering (Gaussian-weighted least-squares straight line fitting, with sigma=50.0s) and physiological noise correction (cardiac and respiratory) using modified RETROICOR (2). The tool *fsl_motion_outlier* was used to remove any time-points that had been corrupted by large motion. The functional images were field-map corrected to reduce B_0_ distortion in the orbital-frontal and temporal regions. Time-series statistical analysis, for both the cold pain and pin prick paradigms, was carried out using FILM (FMRIB’s Improved Linear Model) with local autocorrelation correction.

Higher-level whole brain analysis was carried out using FLAME (FMRIB's Local Analysis of Mixed Effects) stage 1 with automatic outlier detection (3). Z (Gaussianised T/F) statistic images were thresholded using clusters determined by Z>2.3 and a (corrected) cluster significance threshold of P=0.05. A mixed effects analysis (FLAME 1) was conducted in order to generate average activations for each of the contrasts, for the punctate and cold paradigms. Z-score images were generated at a threshold of Z=2.3 with corrected cluster significance of p**<**0.05.

Region of interest (ROI) analyses in the brainstem were conducted using a non-parametric permutation tool, Randomise, which is integrated within FSL. Test statistics were generated from the GLM design and thresholded using threshold-free cluster enhancement (TFCE) and corrected to a p-value of <0.05 (4). Based on the established literature, the brainstem areas selected for ROI analyses were the PAG, nucleus cuneiformis (NCF) and the rostral ventromedial medulla (RVM) (5-9). These areas were masked using hand-drawn anatomical masks referenced from Duvernoy’s Brainstem Atlas.

Post hoc analyses were conducted in order to further investigate the relationship between clinical measures of neuropathic pain severity and change in BOLD signal in brain areas found to show significantly different levels of activation between the nociceptive and neuropathic-like groups.

Functional masks were generated using a 5mm sphere from the peak voxel of activation in the significant cluster of activation corresponding to the rostral anterior cingulate cortex (rACC) identified using whole brain analysis, and from significant clusters of activation detected by the RVM and NCF ROI analyses. Parameter estimates were extracted from the relevant BOLD acquisitions using *Featquery* and the relationship with the mPD-Q score for each patient was interrogated using Spearman’s rank correlation. Finally, these parameter estimates for the rACC and RVM were compared to the post-operative outcomes OKS and the presence of moderate to severe long-term pain after arthroplasty at 12 months post-operatively using Spearman’s rank correlation and Wilcoxon-Mann-Whitney tests respectively.

**Seed-based functional connectivity**

Registration and pre-processing were conducted as described above, except for the application of modified RETROICOR and the *fsl_motion_outlier* tool. De-noising was done using probabilistic independent component analysis, as implemented in MELODIC (Multivariate Exploratory Linear Decomposition into Independent Components) Version 3.15, part of FSL (FMRIB's Software Library, [www.fmrib.ox.ac.uk/fsl](http://www.fmrib.ox.ac.uk/fsl)). Analysis was carried out using Probabilistic Independent Component Analysis (10) as implemented in MELODIC (Multivariate Exploratory Linear Decomposition into Independent Components) Version 3.15, part of FSL (FMRIB's Software Library, [www.fmrib.ox.ac.uk/fsl](http://www.fmrib.ox.ac.uk/fsl)).

The following data pre-processing was applied to the input data: masking of non-brain voxels; voxel-wise de-meaning of the data; normalisation of the voxel-wise variance;

Pre-processed data were whitened and projected into a 42-dimensional subspace using probabilistic Principal Component Analysis where the number of dimensions was estimated using the Laplace approximation to the Bayesian evidence of the model order (10, 11).

The whitened observations were decomposed into sets of vectors which describe signal variation across the temporal domain (time-courses) and across the spatial domain (maps) by optimising for non-Gaussian spatial source distributions using a fixed-point iteration technique (12). Estimated Component maps were divided by the standard deviation of the residual noise and thresholded by fitting a mixture model to the histogram of intensity values (10).

Time-series statistical analysis was carried out using FILM with local autocorrelation correction for anatomical masks of the RVM and rACC, including nuisance regions of interest for cerebrospinal fluid (CSF) and white matter. Higher-level whole brain connectivity of these areas was carried out using FEAT, as described above. Randomise, described above, was used to investigate reciprocal connectivity between the rACC and RVM. In view of the emerging evidence to suggest that pre-exiting aberrant connectivity in the reward system, especially involving the nucleus accumbens (NAc), may contribute to the inability to derive relief from pain-relieving interventions a further post hoc analysis of connectivity between the RVM and NAc was also conducted (13).

Figure 2 Results of mixed-effects analysis of the average group response to punctate, n=24 (A) and cold, n=20 (B) stimuli prior to surgery.


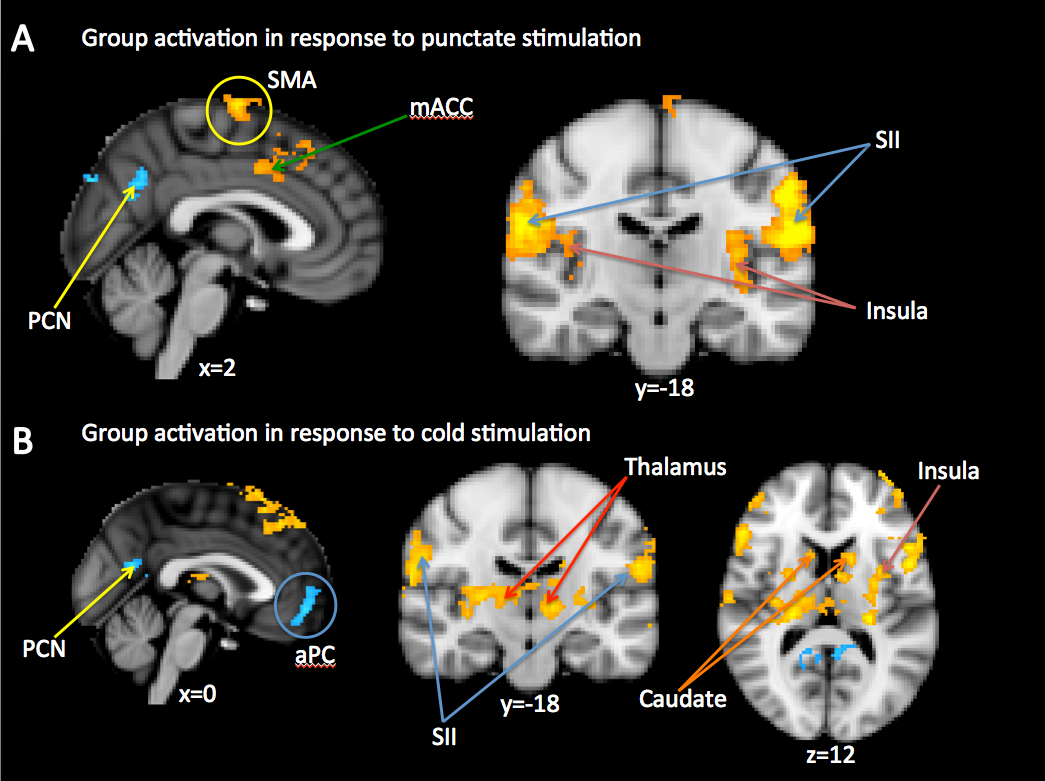


All results are corrected for multiple comparisons (Z score >2.3, P<0.05). Yellow=significantly increased activity, blue=significantly decreased activity. SMA=supplementary motor area; mACC=mid anterior cingulate cortex; SII=secondary somatosensory area; PCN=precuneous; aPC=anterior paracingulate cortex. Images are displayed in radiological convention with MNI co-ordinates given.

Figure 3 Region of interest analysis for the contrast of neuropathic-like > nociceptive, in response to punctate stimulation, and correlation between change in % BOLD signal response and severity of neuropathic-like pain symptoms for the NCF, n=24.


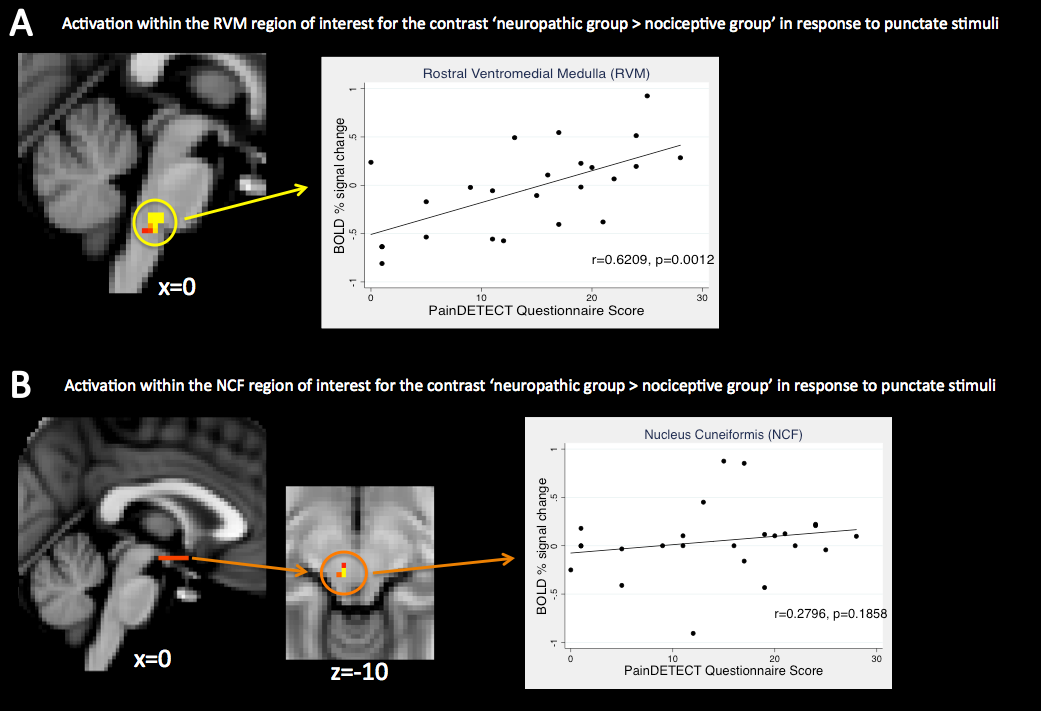


Test statistics were generated from the GLM design and thresholded using threshold-free cluster enhancement (TFCE) and corrected to a p-value of <0.05. Images are displayed in radiological convention with MNI co-ordinates given.

**Figure 4 Relationship between fMRI activation prior to surgery and clinical outcome at 12 months in the rostral anterior cingulate cortex (A), n=19.**

**References**

1. Jesper L. R. Andersson MJaSS. Non-linear optimisation. FMRIB technical report TR07JA1, 2007. 2007.

2. Brooks JC, Beckmann CF, Miller KL, Wise RG, Porro CA, Tracey I, et al. Physiological noise modelling for spinal functional magnetic resonance imaging studies. NeuroImage. 2008;39(2):680-92.

3. Woolrich M. Robust group analysis using outlier inference. NeuroImage. 2008;41(2):286-301.

4. Smith SM, Nichols TE. Threshold-free cluster enhancement: addressing problems of smoothing, threshold dependence and localisation in cluster inference. NeuroImage. 2009;44(1):83-98.

5. Zambreanu L, Wise RG, Brooks JC, Iannetti GD, Tracey I. A role for the brainstem in central sensitisation in humans. Evidence from functional magnetic resonance imaging. Pain. 2005;114(3):397-407.

6. Hemington KS, Coulombe MA. The periaqueductal gray and descending pain modulation: Why should we study them and what role do they play in chronic pain? Journal of neurophysiology. 2015:jn 00998 2014.

7. Gebhart GF. Descending modulation of pain. Neurosci Biobehav Rev. 2004;27(8):729-37.

8. Bee LA, Dickenson AH. Descending facilitation from the brainstem determines behavioural and neuronal hypersensitivity following nerve injury and efficacy of pregabalin. Pain. 2008;140(1):209-23.

9. Ossipov MH, Dussor GO, Porreca F. Central modulation of pain. The Journal of clinical investigation. 2010;120(11):3779-87.

10. Beckmann CF, Smith SM. Probabilistic independent component analysis for functional magnetic resonance imaging. IEEE Trans Med Imaging. 2004;23(2):137-52.

11. Minka T. Automatic choice of dimensionality for PCA. . Technical Report 514, MIT Media Lab Vision and Modeling Group. 2000.

12. Hyvarinen A. Fast and robust fixed-point algorithms for independent component analysis. IEEE Trans Neural Netw. 1999;10(3):626-34.

13. Navratilova E, Morimura K, Xie JY, Atcherley CW, Ossipov MH, Porreca F. Positive emotions and brain reward circuits in chronic pain. J Comp Neurol. 2016;524(8):1646-52.
